# Auditory Processing in Fragile X Syndrome

**DOI:** 10.3389/fncel.2014.00019

**Published:** 2014-02-04

**Authors:** Sarah E. Rotschafer, Khaleel A. Razak

**Affiliations:** Graduate Neuroscience Program, Department of Psychology, University of California, Riverside, CAUSA

**Keywords:** autism, fragile X syndrome, auditory responses, cortex, biomarkers, audiogenic seizures, sensory hypersensitivity

## Abstract

Fragile X syndrome (FXS) is an inherited form of intellectual disability and autism. Among other symptoms, FXS patients demonstrate abnormalities in sensory processing and communication. Clinical, behavioral, and electrophysiological studies consistently show auditory hypersensitivity in humans with FXS. Consistent with observations in humans, the *Fmr1* KO mouse model of FXS also shows evidence of altered auditory processing and communication deficiencies. A well-known and commonly used phenotype in pre-clinical studies of FXS is audiogenic seizures. In addition, increased acoustic startle response is seen in the *Fmr1* KO mice. *In vivo* electrophysiological recordings indicate hyper-excitable responses, broader frequency tuning, and abnormal spectrotemporal processing in primary auditory cortex of *Fmr1 *KO mice. Thus, auditory hyper-excitability is a robust, reliable, and translatable biomarker in *Fmr1* KO mice. Abnormal auditory evoked responses have been used as outcome measures to test therapeutics in FXS patients. Given that similarly abnormal responses are present in *Fmr1* KO mice suggests that cellular mechanisms can be addressed. Sensory cortical deficits are relatively more tractable from a mechanistic perspective than more complex social behaviors that are typically studied in autism and FXS. The focus of this review is to bring together clinical, functional, and structural studies in humans with electrophysiological and behavioral studies in mice to make the case that auditory hypersensitivity provides a unique opportunity to integrate molecular, cellular, circuit level studies with behavioral outcomes in the search for therapeutics for FXS and other autism spectrum disorders.

## INTRODUCTION

Autism spectrum disorder is a growing concern, affecting 0.7–1% of all children born in the United States (Centers for Disease Control and Prevention, 2009). The need for more robust and reliable biomarkers for the symptoms of autism is increasing. Autism is diagnosed through evidence for aberrant social behavior, repetitive behavior, and communication disorder; though evidence of sensory processing anomalies is also present in individuals with autism (Gage et al., 2003a, b; Hitoglou et al., 2010; Marco et al., 2011; Alcántara et al., 2012; O’Connor, 2012; Bhatara et al., 2013). Fragile X syndrome (FXS) is a leading known inherited form of autism, with deficiencies in communication and sensory processing (Largo and Schinzel, 1985; Hanson et al., 1986; Miller et al., 1999; Belser and Sudhalter, 2001; Roberts et al., 2001; Frankland et al., 2004; Price et al., 2007, 2008; Roberts et al., 2007a, b; Barnes et al., 2009). Notably, clinical, behavioral, structural, and functional studies indicate abnormalities in auditory structures and processing in humans with FXS (St. Clair et al., 1987; Rojas et al., 2001; Castren et al., 2003; Van der Molen et al., 2012a, b; Schneider et al., 2013). In 1994, a FXS animal model, the *Fmr1 *KO mouse, was created (Bakker et al., 1994). Consistent with the results in FXS patients, *Fmr1 *KO mice exhibit a variety of abnormal responses to auditory stimuli, and therefore provide a means of studying the neural correlates and mechanisms underlying auditory processing symptoms of human FXS.

## FRAGILE X SYNDROME

Fragile X syndrome is a genetic disorder that affects approximately 1 in 4000 individuals (Hagerman et al., 2009). FXS results from expansion and hyper-methylation of CGG trinucleotide repeats in the promoter region of the *FMR1* gene, which leads to a failure to produce fragile x mental retardation protein (FMRP; Bailey et al., 1998; O’Donnell and Warren, 2002). FMRP inhibits translation of synaptic mRNAs in response to mGluR stimulation, and loss of FMRP typically results in an over-production of associated synaptic proteins (Bear et al., 2004; Bassell and Warren, 2008). Individuals with FXS experience a wide array of symptoms, such as hyper-activity, intellectual impairment, macro-orchidism, hyper-sensitivity to sensory stimulus, and language impairments (Hagerman et al., 1986, 1991; Berry-Kravis et al., 2007; Roberts et al., 2007a; Barnes et al., 2009). Additionally, 10–20% of FXS patients experience seizures (Incorpora et al., 2002; Hagerman and Stafstrom, 2009).

Approximately 15–33% of individuals with FXS meet the three diagnostic criteria for autism, with approximately 5% of autism cases attributed to FXS (Bailey et al., 1998; Cohen et al., 2005). Consistent with autism spectrum disorder, many individuals with FXS display deficits in social behavior, repetitive behavior, and abnormalities in communication. In social interactions, children with FXS often display a “pervasive lack of responsiveness to others” in early childhood, are unwilling to engage in peer play or co-operative play, and generally avoid making eye contact (Hagerman et al., 1986). Also, FXS patients often avoid non-verbal social interactions through a lack of social eye contact, or gaze aversion especially with unfamiliar people or environments (Cohen et al., 1988; Hessl et al., 2006). Individuals diagnosed with FXS may demonstrate a strong preference for routines and a variety of repetitive behaviors, including: rocking, hand-flapping, echolalia, repetitive body movements, and self-injurious behavior (Gillberg et al., 1986; Cohen et al., 1988; Baumgardner et al., 1995; Feinstein and Reiss, 1998; Belser and Sudhalter, 2001; Steinhausen et al., 2002; Baranek et al., 2005).

As with autism, FXS patients show communication abnormalities. Generally, aberrant communication manifests through delays in language development (Fidler et al., 2007; Finestack et al., 2009). Using the Reynell Developmental Language Scales, Roberts et al. (2001) demonstrated delays in communication development in FXS patients manifesting as poor expressive and receptive language skills. Receptive language studies focused on verbal comprehension ability and were assessed through FXS patients’ ability to recognize sound and word patterns. Expressive language was gaged through the breadth of patients’ vocabulary and patients’ ability to verbalize ideas (Roberts et al., 2001). In particular, individuals with FXS experience difficulty articulating words, poor co-articulation, substitutions, and omissions of words, reduction in the number of intelligible syllables produced, difficulty sequencing sounds, and echolalia (Largo and Schinzel, 1985; Hanson et al., 1986; Belser and Sudhalter, 2001; Roberts et al., 2001, 2007a; Barnes et al., 2009). It has been suggested that similar language delays seen in autism may be associated with basic auditory processing abnormalities in early sensory cortical regions (Nieto Del Rincón, 2008; Roberts et al., 2011), but basic auditory processing in humans and mouse models of FXS or autism is incompletely characterized. Taken together, many symptoms of FXS and autism spectrum disorders are similar suggesting that studies of neural alterations in FXS may be broadly applicable.

## NEUROANATOMICAL AND BEHAVIORAL ABNORMALITIES IN FXS

A variety of neuroanatomical abnormalities are seen in FXS patients including a larger caudate nucleus and hippocampus and a reduced superior temporal gyrus (STG), amygdala, anterior ventral cerebral gray matter, and anterior mid-inferior cerebral gray matter (Hessl et al., 2004; Gothelf et al., 2008). Diffuser tensor imaging found alterations in the frontal-caudate and parietal sensory-motor white matter tracts in FXS patients, which may alter the speed of neural processing or the magnitude of neuronal responses (Barnea-Goraly et al., 2003). At the cellular level, a common feature found in the FXS brain is a profusion of abnormally long and thin dendritic spines, with a reduction in the number of short, mushroom-shaped spines (Rudelli et al., 1985; Hinton et al., 1991; Irwin et al., 2002).

Many FXS symptoms may be attributed to over-arching arousal modulation problems, which may underlie the tendency in FXS to avoid sensory experience (Belser and Sudhalter, 1995; Cohen, 1995; Baranek, 2002; Baranek et al., 2002). In a test of electrodermal responses to olfactory, auditory, visual, tactile, and vestibular stimuli, children with FXS showed greater peak amplitude, more peaks, and a failure to habituate to stimuli, suggesting a general over-arousal to sensory stimuli (Miller et al., 1999).

Fragile X syndrome patients demonstrate unusual responses to auditory stimuli specifically, as indicated by clinical studies and as measured by pre-pulse inhibition (PPI; Frankland et al., 2004; Hessl et al., 2009; Yuhas et al., 2011). In PPI tests, subjects are typically presented with a less intense (quieter) pre-pulse stimulus followed by a more intense (louder) startle stimulus. The pre-pulse stimulus acts to suppress the response to the startle stimulus, as most often measured using ocular electromyogram recordings (Frankland et al., 2004; Hessl et al., 2009). Reduced PPI has been found in individuals with FXS but not autism, and individuals with FXS and autism (Frankland et al., 2004; Hessl et al., 2009; Yuhas et al., 2011; Schneider et al., 2012). The magnitude of the PPI response in FXS patients was associated with the severity of symptoms (as measured through IQ, attention span, autism, and adaptive behaviors; Frankland et al., 2004), and the number of CGG repeats. Overall, impaired performance on auditory tests is thought to reflect an underlying problem with sensory gating in FXS.

## AUDITORY PROCESSING IN FXS ASSESSED USING ELECTRO- OR MAGNETO-ENCEPHALOGRAPHY

To assess sensory-cognitive processing in humans with FXS, various event-related brain potential (ERP) techniques have been employed. ERPs reflect the activity of neuronal populations in response to specific sensory-cognitive processes and can be detected using electro-encephalograms (EEG) and magneto-encephalograms (MEG; Luck, 2005). Auditory ERP sensory responses are comprised of N1and P2 components, as well as families of N2 and P3 components (Luck, 2005). The N1 and P2 components are often studied together and can be elicited by simple and complex auditory stimuli, such as pure tones or musical notes (Naatanen and Picton, 1987). Typically, the N1–P2 complex is found within the 80–200 ms following auditory stimulation (Crowley and Colrain, 2004).

The N1 component may be generated by structures within the frontal and temporal lobes (Hari et al., 1982; Naatanen and Picton, 1987). In particular, three basic components have been identified which give rise to a composite N1. There is also MEG and EEG evidence that the auditory cortex is a prime contributor to the N1 component (Zouridakis et al., 1998; Knoth and Lippe, 2012). N1 itself is modulated by the pitch and intensity of auditory stimuli (Beagley and Knight, 1967; Butler, 1968; Pantev et al., 1988; Alain et al., 1997; Butler and Trainor, 2012), and is sensitive to attention effects (Naatanen and Picton, 1987; Luck, 2005; Naatanen et al., 2011a). Specifically, as the intensity of auditory stimulus is increased, the N1 and P2 amplitude increases (Beagley and Knight, 1967; Picton et al., 1970).

Behavioral auditory hyper-sensitivity may result from abnormally increased cortical responses to sound. In six studies using EEG, the N1 component was enlarged in FXS participants (St. Clair et al., 1987; Rojas et al., 2001; Castren et al., 2003; Van der Molen et al., 2012a, b; Schneider et al., 2013). Moreover, there is a reduction in N1 habituation in FXS individuals when presented with repeating trains of single frequency tones (Castren et al., 2003; Schneider et al., 2013). Importantly, the auditory ERP abnormalities can be used as outcome measures in drug treatment in human studies (Schneider et al., 2013). A study using MEG also revealed enlargement and reduced latency of the N100m (the MEG equivalent of the N1 in EEG; Rojas et al., 2001). The N1 amplitude increase may be related to neuroanatomical abnormalities in FXS patients such as related a decrease in STG size (Reiss et al., 1994) and white matter enlargement localized specifically to the temporal lobe (Hazlett et al., 2012). Additionally, fMRI research shows that the STG, along with the medial frontal gyrus, middle temporal gyrus, cerebellum, and pons display higher levels of activation in FXS patients, consistent with the larger N1 component (Hall et al., 2009).

The source of the P2 component has been broadly localized to the temporal lobe, but the specific structures which generate the P2 are somewhat more diffuse (Hari et al., 1980; Knoth and Lippe, 2012). MEG, EEG, and implanted depth electrode evidence suggest that planum temporale and the auditory association cortex (Area 22) are involved in P2 generation (Godey et al., 2001; Crowley and Colrain, 2004). There is also evidence that auditory input to the mesencephalic reticular activating system contribute to the P2 component (Rif et al., 1991; Crowley and Colrain, 2004). P2 amplitude decreases as attention devoted to a stimulus increases (Crowley and Colrain, 2004). Accordingly, the P2 has been suggested to act as an index of task-devoted attention.

Fragile X syndrome-related N1 enhancement is typically accompanied by P2 enhancement as well (St. Clair et al., 1987; Castren et al., 2003; Van der Molen et al., 2012a, b). Because both components seem to stem from temporal lobe activity, it is possible that the structural anomalies and increased temporal lobe activity that likely drives N1 augmentation also contribute to P2. Additionally, P2 enhancement suggests abnormal activation of the mesencephalic reticular activating system, which may contribute to the hyperactivity seen in FXS. Interestingly, alterations in P2 may drive mismatch negativity (MMN), N2b, and P3a abnormalities (Van der Molen et al., 2012b). Structures linked to P2 generation are also responsible for early auditory processing. As such, malfunction of P2-associated structures may create an incorrect memory trace of the target stimulus, which may impair performance on stimulus detection tasks (Naatanen et al., 2007; Naatanen et al., 2011b).

While the N1 and P2 components are readily modulated by altering the spectral components of auditory stimulation, the N2 and P3 component families are generally more heavily involved in task-related selective attention or novelty detection (Breton et al., 1988; Patel and Azzam, 2005). The N2 family is composed of three main components, the N2a/mismatch negativity, the N2b, and the N2c (Patel and Azzam, 2005). N2 components can be elicited with auditory or visual stimulus, and are often probed with an “oddball” paradigm (Patel and Azzam, 2005). Oddball tasks typically involve presenting repetitive trains of a primary stimulus with deviant stimuli interspersed at unpredictable intervals (Breton et al., 1988). N2a, also called MMN, is a feature unique to auditory attention tasks (Cone-Wesson and Wunderlich, 2003). It is associated with bilateral supratemporal processing and right hemisphere frontal lobe activity and is typically seen during tasks that require participants to attend or ignore deviant stimulus (Naatanen et al., 1978; Luck, 2005; Naatanen et al., 2007). Notably, the MMN is present when subjects passively listen to deviant stimuli and when subjects are asked to provide a response to deviant stimuli (Cone-Wesson and Wunderlich, 2003). As MMN is responsive to changes in frequency and intensity of sound, it likely represents the change in attention associated with comparing a deviant tone to the sensory-memory of the control tone (Cone-Wesson and Wunderlich, 2003; Patel and Azzam, 2005; Knoth and Lippe, 2012). The N2b wave can also be generated through oddball tasks, but it is most prominent during voluntary processing of deviant stimuli or when a stimulus is otherwise selectively attended to (Patel and Azzam, 2005). The N2b has also been shown to be modulated by phonological and semantic changes in language (Sanquist et al., 1980). The N2c wave is most strongly associated with visual attention and stimulus context (Folstein and Van Petten, 2008).

Enlargement of the N2b wave (Van der Molen et al., 2012a, b) and increased N2 latency (St. Clair et al., 1987; Van der Molen et al., 2012a) is seen in FXS patients. Despite a general increase in N2 amplitude, the MMN was reduced in individuals with FXS (Van der Molen et al., 2012b). The most likely cause for MMN attenuation is poor memory trace formation of control stimulus (Naatanen et al., 2007). As mentioned earlier, N1 and P2 components are enhanced in FXS. Because N1 and P2 are generated by structures involved in early auditory processing, their aberrant profile may reflect altered perception of auditory stimulus, and therefore an inaccurate representation of the control stimulus (Naatanen et al., 2007). Without an accurate memory trace to compare against deviant tones, individuals with FXS may be less able to identify unexpected stimuli (Naatanen et al., 2007). Interestingly, in studies where participants were asked to respond to, rather than to passively attend deviant stimuli, FXS patients provided more false positives and were slower to respond, suggesting confusion as to the veracity of a stimulus (Scerif et al., 2012; Van der Molen et al., 2012a).

Though the N2b component is typically seen in response to oddball tasks that require participants to attend deviant stimuli, enhancement of N2b may result from a general hypersensitivity to stimuli. In control subjects, the N2b generated in response to the deviant tone was typically larger than the N2b generated by the standard stimulus (Van der Molen et al., 2012b). In FXS subjects, however, there was little difference in the N2b peak amplitudes generated by the deviant and standard stimuli (Van der Molen et al., 2012b). As the N2b peaks generated in response to both the standard and deviant stimuli in FXS participants had greater amplitudes than those of control participants, N2b enhancement in FXS participants may stem from a general increase in sensitivity to any auditory stimuli. Taken together with the reduction in MMN amplitude, oversensitivity to auditory stimulus may impair the ability of FXS participants to discriminate between standard and deviant tones.

The P3 family is comprised of the P3a and P3b components, which are both elicited by infrequent or unpredictable elements introduced into otherwise predictable trains of stimuli. The P3 component typically occurs 300–500 ms after stimulus presentation and is readily evoked with oddball tasks (Hruby and Marsalek, 2003). The source of the P3 component has been localized to the parietal lobe, though there is evidence of hippocampal and temporoparietal structure involvement (Hruby and Marsalek, 2003). The P3a component occurs at 250–280 ms, and is present when infrequent or unpredictable shifts occur during a train of otherwise predictable stimuli regardless of where the participant is asked to direct his or her attention (Squires et al., 1975; Hruby and Marsalek, 2003). As such, the P3a is often described as a “novelty detector” (Comerchero and Polich, 1999; Hruby and Marsalek, 2003). The P3b is also evoked by oddball tasks, and is observed at 250–500 ms (Polich, 2007). Like the P3a, the P3b component is elicited by improbable events. However, the amplitude of the P3b is dependent upon how improbable a stimulus is, with more improbable stimuli resulting in larger amplitude responses (Sutton et al., 1965; Polich, 2007). The P3b response is thought to be distributed across the prefrontal cortex, anterior insula, cingulate gyrus, medial temporal cortex, and hippocampus (Van der Molen et al., 2012a). To gage the predictability of a given stimulus, the ability to recall variations of that stimulus is necessary. As such, short term memory is required for tasks with unpredictable stimuli (Hruby and Marsalek, 2003).

In individuals with FXS, the amplitude of the P3 component was consistently reduced and the latencies to the components were longer (St. Clair et al., 1987; Van der Molen et al., 2012a, b). St. Clair et al. (1987) found a general reduction in P3 amplitude in individuals with FXS, but did not discriminate between P3a and P3b. Van der Molen et al. (2012b) revealed reduced P3a and P3b components in FXS patients. Though the precise source of P3 generation is uncertain, modulation of P3 amplitude or latencies suggest difficulty identifying or responding to infrequent stimuli in FXS patients (Hruby and Marsalek, 2003). Decreased P3b amplitude specifically, may reflect a failure to identify a stimulus as improbable (Sutton et al., 1965), possibly resulting from improper stimulus representation at lower levels of processing, or from short term memory impairments (Polich, 2007). Altered short term memory function may obstruct a FXS patient’s ability to recall a previous stimulus (Polich, 2007). The major auditory ERP abnormalities in humans with FXS are summarized in **Table 1**. 

**Table 1:** Summary of auditory processing abnormalities in humans with FXS determined using auditory ERP.

ERP component	Changes in FXS patients	Reference
N1	Enhanced peak amplitudes, reduced hemispheric asymmetry, reduced habituation	Rojas et al. (2001), Castren et al. (2003), Van der Molen et al. (2012a,b), Schneider et al. (2013)
P2	Enhanced peak amplitude	Van der Molen et al. (2012b)
MMN	Reduced peak amplitude	Van der Molen et al. (2012b)
N2	Enhanced peak amplitude, reduced sensitization, longer latencies	Castren et al. (2003), Van der Molen et al. (2012a,b)
P3	Reduced peak amplitude, longer latencies, abnormal waveform shape	Van der Molen et al. (2012a,b), St. Clair et al. (1987)

## *Fmr*1 KO MOUSE MODEL OF FXS

The *Fmr1* knockout mouse (*Fmr1* KO) was developed as a preclinical model for studying the mechanisms underlying FXS (Bakker et al., 1994). *Fmr1* KO mice lack FMRP and manifest several FXS-associated symptoms (Bernardet and Crusio, 2006; Moy and Nadler, 2008). *Fmr1* KO mice show evidence of social impairments, as demonstrated by social dominance and social interaction tasks (Spencer et al., 2005). Repetitive behaviors have been demonstrated in *Fmr1* KO mice using marble burying tasks (Crawley, 2004, 2007). Through studying ultrasonic vocalization production, evidence has been found that *Fmr1* KO mice experience communication deficits. As pups, *Fmr1* KO mice produce wriggling calls at a higher frequency than their littermate controls, and as adults male *Fmr1* KO mice produce mating calls at a slower rate than male wild type mice (Rotschafer et al., 2012; Roy et al., 2012).

There is also evidence that *Fmr1* KO mice may replicate the heightened anxiety associated with FXS. Elevated plus mazes are a common test for anxiety in mice, but show variable results in *Fmr1 *KO mice. Elevated plus mazes usually have four arms, two of which are enclosed, while two remain open. Reluctance to enter the open arms is suggestive of heightened anxiety, and so fewer entries into open arms can serve as a measure of anxiety (Bilousova et al., 2009). While some studies report *Fmr1* KO mice spend less time in the open arms (Bilousova et al., 2009), others do not (Mineur et al., 2002; Zhao et al., 2005). Open field mazes are used to probe exploratory behavior in *Fmr1 *KO mice. Results of open field maze tasks are also mixed, but do consistently show increased locomotor activity in *Fmr1 *KO mice (Bakker et al., 1994; Mineur et al., 2002). Increased locomotor activity may be related to hyperactivity (Bakker et al., 1994; Mineur et al., 2002). Variability in elevated plus maze tests results may be an artifact of increased locomotor activity or hyperactivity in FXS. Entering the open arms of the plus maze at control levels may reflect *Fmr1 *KO mice moving more vigorously within the maze rather than any anomaly in the anxiety the mouse experiences. The 5-choice serial reaction time task (5CSRTT) specifically tested attentional control while performing an operant task. During the 5CSRTT, mice were presented with an array of five holes in which they may find a food reward. When a light appeared in a hole, mice may select that hole for a reward. The ability of a mouse to accurately choose the illuminated hole measures attention to a task, and the ability of an animal to refrain from choosing a hole prior to predictive stimuli measures inhibitory control. Adult *Fmr1 *KO mice did not demonstrate impairments in accuracy or inhibitory control during testing. However, *Fmr1 *KO mice were hyperactive in novel environments and provided more responses during rule acquisition. During a rule reversal phase, all holes were illuminated and mice had to probe the unilluminated holes to obtain a reward. *Fmr1 *KO mice displayed more errors and a general increase in response rate during the rule reversal phase (Kramvis et al., 2013). These data suggest that hyperactivity in *Fmr1 *KO is largely driven by heightened arousal resulting from exposure to novel environments. Because the neural mechanisms of these complex behaviors are incompletely understood, it is difficult to interpret the actions of potential therapeutics at a circuit level.

## *Fmr*1 KO MOUSE AUDITORY BEHAVIOR

Consistent with auditory abnormalities in humans with FXS, *Fmr1* KO mice show abnormal behavior in response to auditory stimulus, as seen in audiogenic seizure, PPI, and auditory startle response (ASR) paradigms. In *Fmr1* KO mice, intense auditory stimuli (>100 dB SPL) induces a period of wild running, clonic – tonic seizing, and can result in the death of the animal (Musumeci et al., 2000; Chen and Toth, 2001; Musumeci et al., 2007; Dansie et al., 2013). Reintroduction of FMRP to *Fmr1* KO mice significantly reduced audiogenic seizure susceptibility (Musumeci et al., 2007).

The stimulus protocol on mouse PPI studies is similar to that in human testing, with an intense startle stimulus proceeded by a less intense pre-pulse stimulus (Frankland et al., 2004; Bray et al., 2011). *Fmr1* KO mice reliably show enhanced PPI response, a phenotype that is robust enough to be routinely used as a behavioral outcome measure for potential treatments (Olmos-Serrano et al., 2011; Levenga et al., 2011). The PPI response in KO mice is different from humans with FXS, but the respective enhancements and deficits in PPI are both attributed to an underlying aberration in sensory gating (Frankland et al., 2004).

*Fmr1* KO mice show enhanced ASR (Chen and Toth, 2001; Nielsen et al., 2002; Frankland et al., 2004; Yun et al., 2006). Startle enhancement was readily found in *Fmr1* KO mice after 3 weeks of age (Yun et al., 2006). Interestingly, the degree of ASR magnitude was unaffected by stimulus intensity in *Fmr1* KO mice. When wild type mice are presented low intensity stimulus, they respond with relatively small startle response, and relatively large startle responses when presented with high intensity stimulus (Nielsen et al., 2002). By contrast, the magnitude of the *Fmr1* KO mouse startle response did not change with the intensity of the sound stimulus presented (Nielsen et al., 2002). Failure to modulate behavior in response to the magnitude of sensory input is also indicative sensorimotor gating deficits (Nielsen et al., 2002).

## ALTERED *IN VIVO* AUDITORY CORTICAL RESPONSES AND PLASTICITY IN *Fmr*1 KO MICE

Despite the consistent findings of abnormal auditory behaviors and ERPs in humans and hypersensitive responses to sounds in mice, few studies have examined the neural correlates of auditory processing deficits in FXS (Strumbos et al., 2010; Kim et al., 2013; Rotschafer and Razak, 2013). *In vivo* cortical electrophysiological recordings in response to sounds show that individual neurons in *Fmr1* KO mice are hyper-responsive (Rotschafer and Razak, 2013). Mice were presented with a series of pure tone and frequency modulated (FM) sweep stimuli while single unit extracellular recordings were taken in the primary auditory cortex. In response to pure tones, *Fmr1* KO mouse neurons produced more spikes, prolonged responses, and broader frequency tuning than their wild-type counterparts. Neurons from *Fmr1* KO mice also showed more temporal variability in their responses than WT neurons (Rotschafer and Razak, 2013). When presented with FM sweeps, KO neurons that responded best to fast and intermediate FM sweep rates were less sharply selective for sweep rates than those of wild type (Rotschafer and Razak, 2013). Though excessive cortical response to sound may be partially driven by increased activity in other brain nuclei, it is consistent with the abnormal PPI and ASR responses seen in *Fmr1* KO mice as well as with the audiogenic seizure characteristic of *Fmr1* KO mice.

*Fmr1* KO mice also demonstrate mGluR5-associated anomalies in experience-dependent cortical plasticity during the critical period (Kim et al., 2013). An “early” group of *Fmr1* KO mice (P9–P20) and a “late” group of mice (P20–P30) were raised in a sound attenuated chamber while 16 kHz pips were played. Multiunit responses were then used to map frequency representation in the primary auditory cortex of early group, late group, and wild type control mice. Tonotopy in *Fmr1* KO and wild type mice that were not exposed to pure tone stimulus during development was not significantly different. Wild type mice from the early group; however, did demonstrate expanded cortical representation of 16 kHz that the *Fmr1* KO early group failed to replicate, suggesting that cortical plasticity during the critical period may be impaired in *Fmr1* KO mice (Kim et al., 2013). Moreover, neither the wild type nor *Fmr1* KO mice of the late group displayed expanded frequency representation, limiting the window for these plasticity impairments to the critical period. Injections of the mGluR5 antagonist, MPEP, rescued frequency representations in the *Fmr1* KO mouse auditory cortex, implying that heightened mGluR5 activity may underlie this failure of experience dependent plasticity. These data suggest that impaired early developmental plasticity may underlie the abnormalities found in the adult auditory cortical responses (Rotschafer and Razak, 2013). Such cortical deficits may lead to higher order auditory processing deficits such as those involved in language. The major auditory behavioral and processing abnormalities in the *Fmr1* KO mice are summarized in **Table 2**. 

**Table 2:** Summary of auditory processing abnormalities in the *Fmr1* KO mice.

Auditory deficit	Study method	Reference
Increased propensity for audiogenic seizures	Behavioral response to loud sounds	Musumeci et al. (2000, 2007), Chen and Toth (2001), Dansie et al. (2013)
Increased acoustic startle response	Behavioral observations	Chen and Toth (2001), Nielsen et al. (2002), Frankland et al. (2004), Yun et al. (2006)
Altered auditory cortical responses to sounds Increased and prolonged single neuron responses to pure tones	*In vivo* extracellular electrophysiology	Rotschafer and Razak (2013)
Broader frequency tuning curves
Reduced spectrotemporal selectivity
Increased variability in response latency
Reduced critical period plasticity in auditory cortex	Sound exposure during development and *in vivo* electrophysiological mapping	Kim et al. (2013)
Altered tonotopy and reduced experience dependent plasticity of Kv3.1b channel in auditory brainstem	Sound exposure, immunohistochemistry and electrophysiology	Strumbos et al. (2010)

## ALTERED CORTICAL EXCITATORY-INHIBITORY BALANCE MAY UNDERLIE ABNORMAL AUDITORY RESPONSES

The enhanced single neuron responses in the auditory cortex is consistent with a general increase in the excitability of neurons lacking FMRP, and subsequently, over-active neuronal networks. These responses may be neural correlates of auditory hypersensitivity in humans with FXS, and thus provide functional probes to investigate the underlying receptive field and molecular mechanisms in the mouse model. The heightened activity may stem from a concurrence of dysfunctional intrinsic excitability and GABA receptors, loss of inhibitory neurons, abnormal dendrite morphology, and excessive mGluR activity (Huber et al., 2002a, b; Huber, 2007). For example, neurons in the *Fmr1* KO mouse somatosensory cortex show weakened inhibitory interneuron activity and more excitable pyramidal neurons (Gibson et al., 2008; Hays et al., 2011; Paluszkiewicz et al., 2011). Monosynaptic GABAergic transmission in the barrel cortex of *Fmr1* KO mice is unaffected, but fast spiking (inhibitory) interneurons experience an approximate 50% decrease in excitatory drive (Gibson et al., 2008). *Fmr1* KO mouse somatosensory neurons are also hyperexcitable, as characterized by longer, less synchronous UP states (Gibson et al., 2008; Hays et al., 2011). UP states were prolonged in cortical slices that did not express *Fmr1 *in glutamatergic neuron, while deletion of *Fmr1 *in GABAergic neurons had no effect on UP state duration (Hays et al., 2011). Prolonged UP states are generated by the over activity of mGluR5 receptors on excitatory neurons which lack *Fmr1*, rather than any *Fmr1 *deficiency in inhibitory neurons (Hays et al., 2011). There might also be abnormalities at the cortical network level as a result of single neuron changes. For example, young (first two post-natal weeks) *Fmr1 *KO mice show increased network synchrony when spontaneous firing was assessed with two-photon Ca^2^^+^ imaging (Gonςalves et al., 2013). In *Fmr1 *KO mice, increased network synchrony was strongly correlated with increased action potential production (Gonςalves, 2013).

Consistent with behavioral research demonstrating mGluR antagonists and GABA receptor agonists improve *Fmr1* KO mouse responses to auditory stimuli, evidence of both GABA and glutamate imbalances have been found in *Fmr1* KO mice. The mRNA of the α_1_, α_3_, α_4_, β_1_, β_2_, γ_1_, and γ_2_ GABA receptor subunits are down-regulated by 35–50% in *Fmr1* KO mice, with a decline in the actual α_1_, β_2_, and δ GABA receptor subunits (El Idrissi et al., 2005; D’Hulst et al., 2006, 2009; Adusei et al., 2010). Additionally, a 20% reduction in the number of Parvalbumin positive (PV+) cells was found in the somatosensory cortex of *Fmr1* KO mice (Selby et al., 2007). Underlying alterations in GABA receptor function and excessive excitatory input may result in some changes in auditory behavior seen in *Fmr1 *KO mice.

Like FXS patients, *Fmr1* KO mice exhibit abnormal cortical and hippocampal dendritic spine morphology. Dendritic spines on pyramidal cells in the visual and temporal cortex of *Fmr1* KO mice are longer, thinner, and generally display a more immature morphology than wild-type mice, with increased spine density along dendrites (Comery et al., 1997; Irwin et al., 2000, 2002; McKinney et al., 2005). The *Fmr1* KO mouse barrel cortex has been studied more extensively and shows a host of dendritic spine abnormalities. Relatively young mice (P25) barrel cortex did not show dendritic spine abnormalities, but older mice (P73–P76) show fewer short/ mature spines and more long/immature spines (Galvez and Greenough, 2005; Till et al., 2012). Transcranial two-photon imaging revealed that dendritic spines in the barrel cortex of *Fmr1* KO mice also display a higher turnover rate, with more pools of new, transient spines (Pan et al., 2010).

The *Fmr1* KO mouse barrel cortex also demonstrates delayed formation, and abnormal dendrite pruning. In mice, each vibrissa (whisker) is represented by a cortical barrel that has a cell body dense septa and cell-sparse hollow. During development, the number of dendrites at the septa decreases, while the number of dendrites growing to the hollow increases. Pruning of dendrites growing toward the septa results in asymmetrical dendrite distribution in wild type adult animals (Greenough and Chang, 1988). In *Fmr1* KO mice, spiny stellate cells in the barrel cortex have an excessive number of dendrites oriented toward the septa, resulting in less asymmetrical cells (Galvez et al., 2003; Till et al., 2012). Barrel cortex neurons of *Fmr1 *KO mice also show excessive dendritic spine production and unusually long spines during the first post-natal week. When dendritic spines were examined at post-natal weeks 2 and 4, a trend toward normalization was discovered. While dendritic spines were still longer in *Fmr1 *KO mice, the magnitude of difference from wild type animals had decreased. The number dendritic spines in *Fmr1 *KO mice had fallen to numbers comparable to WT animals by the second week (Nimchinsky et al., 2001). Disrupted cell morphology and cytoarchitecture during early development of sensory systems may impair an animal’s ability to integrate sensory experiences. Functionally, dendritic spines in the *Fmr1* KO mouse barrel cortex are less sensitive to sensory experience modulation. Sensory deprivation (all whiskers on one side of the facial pad were trimmed) resulted in a reduced the rate of dendritic spine elimination in wild type mice, but was unaltered in *Fmr1* KO mice (Zuo et al., 2005; Pan et al., 2010). Alternatively, dendritic spine formation was enhanced in wild type mice when the whiskers were trimmed in a chessboard pattern, while *Fmr1* KO mice failed to show any difference in spine formation. Failure to form or eliminate dendritic spines in response to changing sensory input suggests that barrel cortex neurons in *Fmr1* KO mice may be improperly tuned to sensory stimuli (Pan et al., 2010). A similar pattern if found in the auditory cortex may explain the reduced developmental plasticity (Kim et al., 2013) and abnormal adult responses (Rotschafer and Razak, 2013).

## CONCLUSIONS AND FUTURE STUDIES

Converging lines of evidence describe auditory cortical dysfunction in the *Fmr1* KO mice and in patients with FXS. The common underlying phenotype is “auditory hypersensitivity.” The mechanisms underlying sensory hypersensitivity may be relatively more tractable compared to more complex social behaviors typically studied in FXS (and autism). Therefore, we propose that auditory hypersensitivity is a robust, reliable, and translatable biomarker to integrate pre-clinical and clinical investigations at multiple levels of analysis to facilitate drug discovery in FXS. Within this framework, the following future studies will be important to perform:

(1) What are the neural mechanisms of individual neuron hyper-excitability in the primary auditory cortex of the* Fmr1* KO mice? Understanding the intrinsic properties, morphological features, and inhibition/excitation balance in auditory cortical neurons is a necessary step in understanding the mechanisms of auditory hypersensitivity in mice and humans. The availability of mice in which FMRP can be deleted specifically in the cortex, in specific types of neurons and at specific developmental time points will make it possible to identify the loci of hyper-excitability.
(2) Fragile X syndrome is a neurodevelopmental disorder, but the developmental changes in functional neural response selectivity are unclear. The focus has been on transient delays or permanent changes in dendritic spine properties and synaptic or intrinsic properties (reviewed in Meredith et al., 2012). The consequence of these synaptic changes to development of behaviorally relevant neural response properties are not known in FXS. Therefore, it is not clear if the observed auditory hypersensitivity in humans with FXS is due to an altered regulation of developmental processes during a sensitive period that persists into adulthood. The majority of studies using *Fmr1* KO mice focus on the neurological responses and behaviors of relatively young mice, neglecting any long term developmental changes *Fmr1* KO may undergo and failing to describe the long term impact of the dysfunction described early in life. Are the results of FMRP loss during a specific sensitive period in life or whether they may better be attributed to the on-going absence of FMRP? It will be useful to determine if there are specific time developmental windows during which the KO and WT mice cortical responses diverge from each other. As summarized by Meredith et al. (2012), it may be possible to target specific developmental windows for therapeutic approaches. Auditory hypersensitivity will provide translatable physiological and behavioral probes to address this possibility and study underlying mechanisms.
(3) Development of methods to monitor long term auditory processing in awake behaving mice. One such technique may be chronic EEG from the temporal cortex of WT and KO mice. This method will not only provide baseline and sound evoked EEG, but also facilitate a study of neural activity during audiogenic seizure induction. Because audiogenic seizure is a commonly used behavior in testing drugs for FXS, having the additional measure of electrical activity during seizure induction, will provide a rich set of biomarkers to measure drug effects. Moreover, the evoked EEGs will allow a more direct comparison with human auditory ERP studies, facilitating translation efforts.

## Conflict of Interest Statement

The authors declare that the research was conducted in the absence of any commercial or financial relationships that could be construed as a potential conflict of interest.
